# Remote assessment and practice implementation for disorders in ears, nose and throat: a mixed-methodology observational study in the United Kingdom

**DOI:** 10.1136/bmjopen-2025-115521

**Published:** 2026-04-15

**Authors:** Dimitrios Spinos, Thomas Beech, Christopher Coulson, Sheila Greenfield, Ian Litchfield, Paul Nankivell, Richard Allen, Jameel Muzaffar

**Affiliations:** 1 Department of Cancer and Genomic Sciences, University of Birmingham, Birmingham, UK; 2 Department of Ear, Nose and Throat Surgery, University Hospitals Birmingham, Birmingham, UK; 3 Institute of Applied Health Research, University of Birmingham, Birmingham, UK; 4 The Open University, Milton Keynes, UK

**Keywords:** Digital Technology, OTOLARYNGOLOGY, Waiting lists

## Abstract

**Abstract:**

**Introduction:**

Ear, nose and throat (ENT) conditions are highly prevalent in primary and secondary care, yet patients frequently face prolonged waits for specialist review. In England, over half of ENT patients wait beyond the NHS 18-week referral-to-treatment target. Many of these cases can be effectively managed with advice and non-surgical interventions, presenting an opportunity for remote service innovation.

This study aims to evaluate the clinical effectiveness, cost-effectiveness, acceptability and environmental sustainability of a digitally enabled remote ENT clinic model compared with traditional face-to-face pathways.

**Methods and analysis:**

This single-centre, mixed-methods, prospective cohort study will be conducted at University Hospitals Birmingham NHS Foundation Trust. Remote clinics will use trained staff to collect diagnostic data (including endoscopic imaging and boothless hearing tests) for consultant review via secure cloud-based software. Quantitative analysis will assess patient outcomes, costs, waiting times, carbon footprint and satisfaction. Qualitative data from semi-structured interviews with patients, clinicians and managers will explore acceptability, scalability and barriers to implementation. The qualitative data will be analysed using the framework methodology, according to the non-adoption, abandonment, scale-up, spread and sustainability framework, while the strengthening the reporting of observational studies in epidemiology framework will be used to guide the reporting of quantitative data. Cost-effectiveness analyses will follow NICE guidelines, while environmental impact will be measurement will be informed by the sustainability in quality improvement framework. Recruitment will be aiming for 300 completed datasets and 30–35 interviews.

**Ethics and dissemination:**

Ethical approval has been granted (IRAS 350908; REC 25/SW/0116). Findings will be disseminated via conferences, peer-reviewed journals and institutional communication channels.

**STRENGTHS AND LIMITATIONS OF THIS STUDY**

- Mixed methodology analysis: holistic capture of different types of data reflecting the wider impact and ramifications of the implementation of remote specialist clinics.
- Real-world implementation: data is captured in real-world settings, allowing study of the challenges of providing a service away from a controlled environment.
- Study site characteristics: the study sites offer hundreds of ear, nose and throat clinical encounters per week, facilitating data capture for a high volume and diverse population of patients.
- Selection bias: the study takes place in a single multi-centre NHS Trust, and therefore runs a risk of potentially excluding patients of particular demographics that do not attend that Trust.

## Introduction

 In 2009 the NHS Constitution set the standard of 92% of patients waiting no longer than 18 weeks from referral to treatment.^1^ In December 2024, 7.5 million patients in England were waiting for an appointment, with more than two-fifths waiting beyond the NHS standard.^2^ That proportion varies by speciality, with ear, nose and throat (ENT) demonstrating the worst waiting times, with over half of the patients exceeding the 18-week target.^1^

Hearing and ear problems, alongside nasal symptoms, are common in primary and secondary healthcare, with ear pain, discharge, recurrent infections, chronic rhinosinusitis and allergic rhinitis particularly common. While primary care management may suffice, many patients need specialist ENT referrals for diagnosis and management. However, lengthy waits, often exceeding twelve months, are commonplace for specialist consultations.^3^ This equates to over half a million patients having increased time spent with disease, distress and also potentially a poorer prognosis. Recent evidence suggests ENT patients are more commonly managed with advice, medications and then discharge, when compared with specialities such as orthopaedics or gynaecology with a higher proportion converting to surgical intervention,^4^ indicating that they can be effectively managed with fewer appointments and occasionally, entirely remotely.

Responding to the delays facing patients, a digitally empowered, remote ENT system has been developed by our team of collaborators, capitalising on advances in endoscopic nasal and ear imaging and boothless hearing tests. A pilot study, comprising 53 patients, demonstrated that 83% of cases could be effectively managed remotely via this service, with high levels of satisfaction reported by both patients and general practitioners.^5^

**Figure 1:**
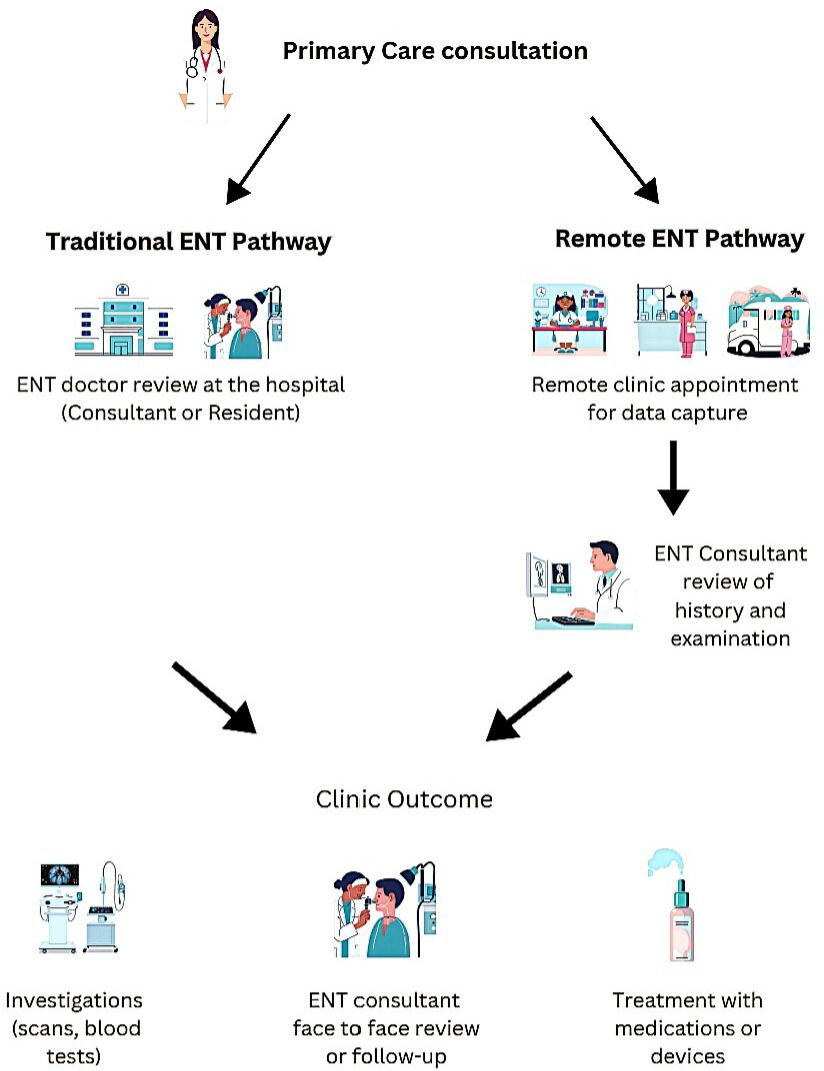
Traditional and remote clinic pathways infographic. ENT, ear, nose and throat.

The remote service uses trained nursing or physician associate staff to ‘collect’ the necessary patient data, for example, supervision of tablet history collection and boothless audiogram, and perform video of patient ears/nose, thereby allowing consultants to review this information and diagnose and manage patients remotely (figure 1). Audiometric testing will be performed using calibrated boothless audiometers developed by hearX Group (Pretoria, Gauteng, South Africa). These devices are commercially available systems that meet the required regulatory and calibration international standards for clinical use (ISO 8253-1/ISO 389 Series/IEC 60 645-1/ANSI S3.6) and are CE-marked registered and certified in Europe. The company’s regulatory standards are available in detail on their webpage: https://www.hearxgroup.com/products/heartest. This enables optimisation of resources through face-to-face, technician-delivered clinics in varied locations with consultants providing opinions flexibly. The service can be placed in different locations, closer to the patients, reducing travel and environmental impact. The University Hospitals Birmingham (UHB) NHS Foundation Trust has approved the introduction of this novel pathway into their outpatient referral pathway from primary to secondary care, currently facilitating the delivery of Otology and Rhinology clinics.

Our team has conducted a review of the literature to inform and guide the design of this study.^6^ Despite evidence supporting the feasibility and safety of digitally enabled remote ENT clinics, no consensus exists on the ideal placement of such a service. Moreover, there is still a need to further understand this pathway’s real-world effectiveness and acceptability across different stakeholders (patients, clinicians, managers), compared with the traditional face-to-face consultation model. Moreover, we need to investigate whether there are any specific groups of patients that such a digitally empowered pathway would work better for compared with others. Furthermore, aligning with the NHS net-zero agenda, we need to understand whether RAPID ENT could contribute to the reduction of the carbon footprint of the ENT outpatient care delivery.

Our research focus will evaluate the clinical and cost-effectiveness, acceptability and environmental sustainability of the remote clinics model, compared with the traditional pathway. This study is due to launch in October 2025 and will be undertaken for a period of up to 2 years, with October 2027 being defined as the end date of the study. The end of the study will be the date of the last follow-up and data collection via questionnaires and interviews of patient-participants, post the completion of their treatment. Documents will be archived following the University of Birmingham (UoB) Code of Practice for Research.

## Methods and analysis

### Project setting

The study will be conducted across hospital and primary care outpatient clinics offered by UHB. Data will be collected from patients following all permutations of the remote pathway alongside standard ENT outpatient clinics, such as hospital outpatients, diagnostic hubs and primary care clinics. During appointments, patient data captured via the Endoscope-I software will be stored securely. Post-consultation, data will be transmitted through an encrypted cloud service and reviewed by ENT Consultants using secure, bespoke app-based software. Following completion of their outpatient pathway, consented patients will be asked to complete a follow-up questionnaire, and some will be invited to a semi-structured interview.

### Primary outcome

#### Clinical efficiency

Assess the proportion of patients who can be successfully diagnosed and managed remotely and calculate the number of appointments required to reach definitive treatment. We define “successful remote management” as patients with a diagnosis and treatment plan decided after a single remote consultation, without further appointments required to reach diagnosis/initial management. Further follow-up appointments that were required as part of further investigations or treatment do not constitute a “failed remote management” unless diagnosis was not reached after the initial appointment. Assess re-presentation rates to the ENT services for patients that attended the remote clinics.

### Secondary outcomes

1. Clinical throughput impact:
  - Investigate impact of remote pathway on waiting times from referral to treatment.
  - Compare the clinician time required per remote consultation to reach diagnosis/definitive treatment.
2. Stakeholder acceptability:
  - Examine barriers to implementation, training needs, scalability and throughput.
  - Determine the optimal setting for data capture and assess geographic variation.
  - Assess the impact across different demographics, especially under-served populations.
3. Health economic analysis:
  - Conduct a cost-consequences analysis detailing waiting times, quality of life, resource use, costs, time off work and productivity for both study groups.
  - Perform cost-consequence and cost-utility analyses from an NHS perspective.
4. Environmental sustainability:
  - Calculate the direct (patient journey) and indirect (clinic output) carbon footprint of the ENT outpatient service by assessing carbon dioxide equivalents (CO_2_Eq) for both new and existing clinic models by location.

### Theoretical framework

The remote pathway implementation will be informed by the non-adoption, abandonment, scale-up, spread and sustainability (NASSS) framework, developed to support, guide and monitor the implementation of digital technologies in health and social care.^7^ The NASSS framework uses seven domains to describe various aspects of the technology, end users, organisational structure and broader policy-driven context.^8^ The principles of a single centre prospective cohort study will be followed according to the STROBE statement^9^ for quantitative data analysis. We will measure the environmental impact of the remote clinic service, the Centre for Sustainable Healthcare’s sustainability in quality improvement (SusQI) framework^10^ that aligns with our aim of embedding principles of sustainable practices within healthcare.

### Inclusion criteria

Patient participants: patients referred from primary care, adults (18 years of age or older), with conditions of the ears or nose.

Non-patient participants: stakeholders with previous or current experience in digital or service transformation, stakeholders engaging in the implementation or delivery of the remote-enabled ENT clinical service.

### Exclusion criteria

Patient participants: patients referred for tertiary assessment, and patients with suspected cancer, patients younger than 18 years old. Patients with balance or vertigo problems as their primary presentation.

Non-patient participants: stakeholders without previous or current experience in remote services, stakeholders without understanding or knowledge of ENT clinic service delivery or design.

### Recruitment

Patients referred from primary care to ENT specialist services and attending either a conventional face-to-face consultant-led clinic or a remote clinic will be recruited into the study. We will send an invitation letter explaining the purpose of the study to the patients, along with their clinic invitation letter, and potential participants will be approached prior to their initial clinic appointment.

On their attendance at clinic, patients will be approached by a member of the healthcare team and will be given the study information package and offered the opportunity to ask questions and discuss their potential participation in the study. For those patients who agree to take part, this will be documented in their medical records and the research team will begin informed consent procedures.

Patients will not be randomised, and the study will not have any influence on the allocation of patients to the traditional or the remote pathway, which will be arranged by UHB, on a weekly basis, according to clinical priority and waiting length per referral. The clinic allocation process is regulated by the hospital and patients are allocated in consecutive order to either a face-to-face or a remote clinic, until all the slots of that week have been filled, before booking patients for a different week. Patient prioritisation is made in the form of urgency, without reference to the method of clinic delivery. Patient demographics, disease severity or other characteristics are not used in regards to allocation to an in-person or remote clinic appointment.

This will ensure that selection bias will not be introduced on the patients seen in the remote pathway, compared with the cohort seen face-to-face. We will assess propensity scores for the patients and assess their propensity score weighting using logistic regression.

This is an observational study, and we are not measuring the effect of an intervention. Our initial sample size calculations have been driven by similar pieces of implementation studies in the literature, looking into studies assessing non-inferiority interventions for remote/telehealth clinics, as well as assessing the potential financial benefit to the patients/service and the environmental impact,^11^,^15^ further informed by similar studies mapped through our team’s scoping review.^6^ Continuous sampling will be undertaken on both pathways until we reach a number of 150 participants from each group that have completed the initial questionnaires. The patients of the remote pathway will also be sent a follow-up questionnaire after their discharge, asking for their overall experience of the new service. The total of recruited participants will be expected to exceed the minimum of 300 we need for our analysis, and we will be allowing for a 10% attrition, as the current number for patients seen in face-to-face and remote clinics exceeds the 80 patients per cohort, per week. All the primary outcomes data will be extracted directly from the clinical notes of the patients, while the data of the secondary outcomes will be procured via questionnaires.

For the interviews, we will use a topic guide developed based on the existing literature and the research team’s experience of technology and innovation implementation, as well as service redesign.^16^,^18^ Through the interview process, we will investigate the experience of the different stakeholders involved in the remote pathway. We will undertake purposive sampling to select participants, expecting 30–35 participants in total, to allow for thematic saturation to take place.^19 20^ Participants in the interviews will be classified into two categories: patient participants and NHS staff. Recruitment will continue until overall thematic saturation has been achieved within each group. Patients enrolled will be approached, after completing the remote clinic pathway, while clinicians, managers and the other stakeholders will be given participation leaflets and invited via email or in person to participate in the study through interviews. If non-English speakers participate in the interviews, interpreters will also be facilitating the process.

### Quantitative methodology

**Figure 2:**
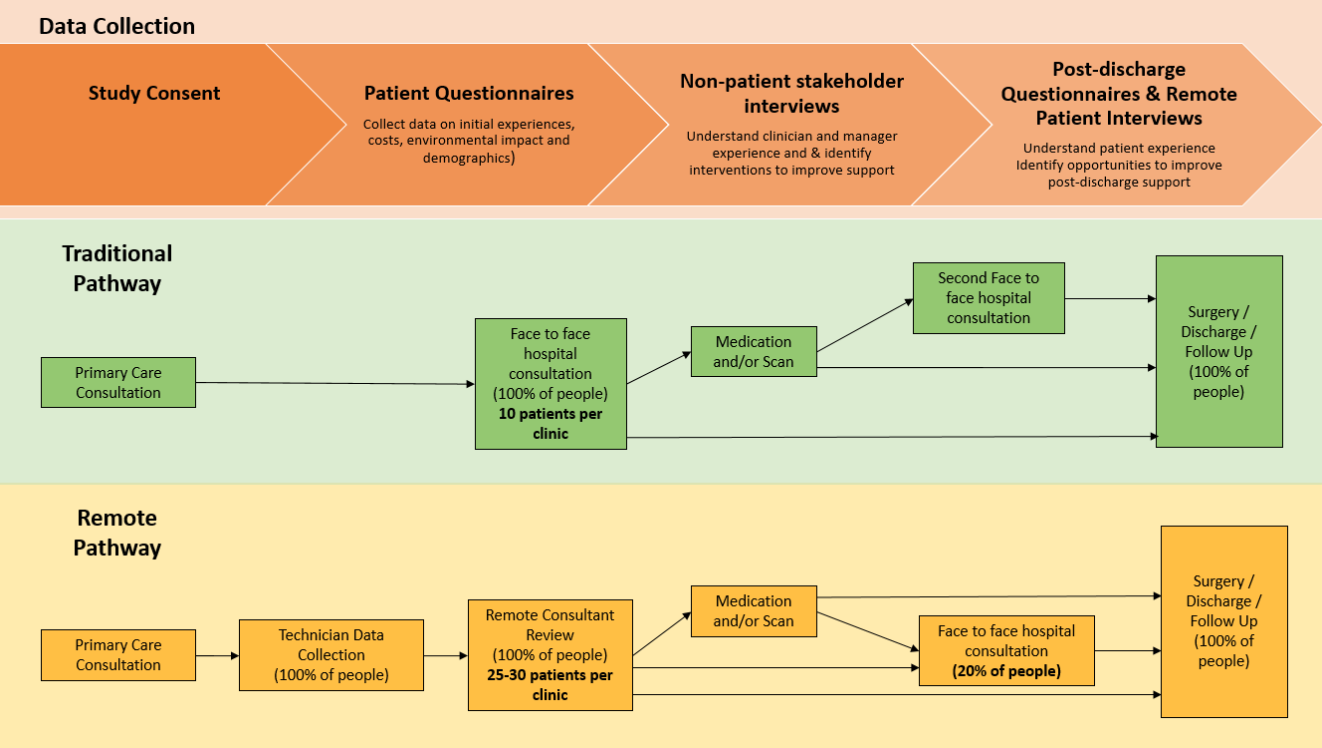
Traditional and remote clinics and data capture points.

Quantitative methodology study process using questionnaires (figure 2):

- Patients attending traditional pathway and new remote pathway recruited.
- Remote pathway participants: two questionnaires - first on the day of clinic attendance and second after completion of treatment or discharge.
- Traditional pathway participants: one questionnaire on the day of clinic attendance.

### Quantitative analysis

Quantitative analysis will examine patient characteristics, symptom resolution, discharge rates, consultant review volumes and waiting times, as well as environmental sustainability (assessed by the carbon footprint of clinics and patient travel).

Acceptability will be gauged using a 5-point Likert scale and open-ended questionnaires. Descriptive statistics (means, SD and CIs) will summarise continuous data, while discrete variables will be reported as counts and percentages. Standard regression models (95% CIs) and subgroup analyses will compare groups, with outcomes reported in scoring scales analysed as continuous variables. Count models will be used to count outcomes (such as number of appointments) and time-to-event models to assess time to definitive treatment/decision. A 5-point Likert scale questionnaire will further capture how different clinic permutations align with patient expectations. Missing data will be handled initially by descriptive means, aiming to describe the proportion of missing data, its characteristics and potential mechanisms and then using multiple imputation methodology. Analyses will adjust for age, sex, IMD quintile, baseline symptoms, presenting condition category, comorbidity count and distance to clinic; robustness will be checked using propensity score weighting. Differential follow-up will be handled with time-to-event methodology, using Cox proportional hazards, accounting for the differences in the follow-up duration across groups with different pathology.

### Qualitative methodology

Qualitative methodology study process using semi-structured interviews (figure 2):

- 1:1 interview with patients, clinicians, managers and other stakeholders involved in the remote ENT pathway.
- Interviews conducted on Microsoft Teams with audio recording for transcription.
- Analysis.

### Qualitative analysis

MS Teams software provides auto-transcription of the interviews and NVivo software will be used to facilitate the data management. Professional translation will be available for non-English speakers and manual transcription will be undertaken if necessary. The data will be analysed using a directed content analysis to populate the NASSS framework using open matrix approach, as described by Elo *et al* and Assarroudi *et al*^21 22^ that will allow for the inclusion of emerging constructs within existing domains, including any project specific risks/challenges and opportunities identified by interviewees in the different groups. Any novel emergent themes will be recorded and coded into separate categories, allowing the classification of relevant information in the respective groups. We will compare themes within each group and between groups.

Two researchers (DS and IL) will independently allocate data within the framework. Any differences in coding will be discussed between the authors (DS, IL and SG) and a consensus will be arrived at. The final allocation of the data within the NASSS framework will be agreed by all authors. Rigour will be further enhanced via a reflexivity statement by the researchers involved in the coding and analysis. Qualitative data will be reported according to the consolidated criteria for reporting qualitative research checklist.^23^

### Health economic methodology and analysis

A comprehensive health economic analysis will assess the cost-effectiveness of the remote model across settings. This will include an examination of NHS costs for training, equipment and service delivery (current tariffs), alongside economic benefits derived from improved patient outcomes and system efficiencies. Productivity will be assessed from both NHS and patients’ perspectives. Health utility will be assessed via PROMs and successful treatment/discharge from ENT care. Cost-consequence and cost-utility analyses will be undertaken in line with NICE guidelines.^24^ Data will be standardised and allocated to clinical pathways, with incremental cost-effectiveness ratios generated to compare models. Results will be summarised descriptively for each cost category, highlighting any pathways with a significant impact on overall costs or outcomes.

### Environmental sustainability methodology and analysis

The environmental impact of the service will be assessed using CO_2_eq and estimates of direct waste, including that arising from patient travel. Using the SusQI framework, CO_2_eq will be mapped across the different clinic iterations, with standard regression analysis used to compare the carbon output of RAPID ENT setups with that of traditional clinics. CO_2_eq will include patient travel, clinic energy use (capture booth/otoscope/endoscope, computing) and consumables. Distance will be calculated by patients’ estimates and postcode. Emission factors will be drawn from current UK Government conversion factors; uncertainty will be propagated via non-parametric bootstrap.

### Patient and public involvement

Patient and public involvement and engagement (PPIE) group has been a crucial component of this study, and a previous dedicated group contributed to the initial design of the current project plan and project aspirations, in line with the GRIPP2 guidance.^25^ The study-specific group that has been employed since the initial iterations of this study consisted of members of the UHB rhinology and audiology PPIE groups, while external contribution from the “1000 Elders” UoB PPIE group, using focus groups. They have been involved in reviewing our proposal, helping us identify the patients’ priorities and what accounts to a “successful” clinical encounter to them, as well as contributing to the final version of this protocol. Following the principles of co-design and co-production, the PPIE lead contributed to previously published work on the application of PPIE groups in ear and hearing research, a field with no previous published literature.^26^

## Ethics and dissemination

UoB is the sponsor of this study (study number RG_25-016) and this study received ethical approval by the Health Research Authority on the 2nd of October 2025 (IRAS 350908, REC reference 25/SW/0116), contact email: contact@hra.nhs.uk. This study has been publicly registered in the Open Science Framework database: https://doi.org/10.17605/OSF.IO/BRQC3.

Results will be disseminated more widely by presentation at national and international conferences, such as the Confederation of European Otorhinolaryngology, Head and Neck Surgery Conference, and publication in open access, peer-reviewed journals. We will also communicate our findings through the social media sites of UoB and UHB.

### Data availability

Data are available upon reasonable request. De-identified participant data will be stored in UHB and UoB cloud. Requests for access can be made through the corresponding author and upon review by the sponsor of the study, access and reuse will be considered.
