# Supplementary material for: Remote assessment and practice implementation for disorders in ears, nose and throat: a mixed-methodology observational study in the United Kingdom
Source: BMJ Open. 2026 Apr 15;16(4):e115521. doi: 10.1136/bmjopen-2025-115521 (PMC13084778; doi:10.1136/bmjopen-2025-115521)
Supplement: Supplementary file 1 [file bmjopen-2025-115521.reviewer_comments.pdf]

PEER REVIEW HISTORY

BMJ Open publishes all reviews undertaken for accepted manuscripts. Reviewers are asked to complete a checklist review form and are provided with free text boxes to elaborate on their assessment. These free text comments are reproduced below.

ARTICLE DETAILS

Title (Provisional)

Remote Assessment and Practice Implementation for Disorders in Ears, Nose and Throat: a mixed methodology observational study in the United Kingdom

Authors

Spinos, Dimitrios; Beech, Thomas; Coulson, Christopher; Greenfield, Sheila; Litchfield, Ian; Nankivell, Paul; Allen, Richard; Muzaffar, Jameel

---

VERSION 1 - REVIEW

**Reviewer**	**1**
**Name**	**Fu, Xinxing**
**Affiliation**	**Beijing Tongren Hospital, Beijing Institute of Otolaryngology**
**Date**	**01-Feb-2026**
**COI**	**None**

This protocol addresses a highly relevant healthcare challenge by evaluating a digitally enabled remote ENT clinic model in terms of clinical efficiency, cost-effectiveness, acceptability, and environmental sustainability. The mixed-methods design is appropriate for capturing both system-level outcomes and stakeholder experiences, and the use of established frameworks such as NASSS, STROBE, NICE guidance, and the SusQI framework strengthens the theoretical and methodological foundation of the study.

Overall, the proposed research is timely, well motivated, and aligned with current NHS priorities regarding waiting list reduction and sustainable healthcare delivery. However, several methodological aspects would benefit from further clarification to enhance transparency, reproducibility, and the validity of future interpretations.

First, the protocol refers to the use of “boothless hearing tests” as part of the remote diagnostic pathway, yet no information is provided regarding the specific technology used, its validation status, or its agreement with standard audiometric testing performed in sound-treated environments. Given the central role of hearing assessment in ENT care, clearer description of the measurement methods and supporting evidence for their clinical reliability is essential.

Second, although patients are not randomised to the remote or traditional pathways, the manuscript states that allocation based on clinical priority and waiting time will not introduce selection bias. In practice, these factors are likely to be associated with disease severity, comorbidity, and sociodemographic characteristics, which may influence both outcomes and service utilisation. Further explanation of how pathway allocation decisions are made, and how potential confounding will be addressed beyond propensity score weighting, would strengthen the methodological rigour.

Third, the primary outcome of “successful diagnosis and management remotely” would benefit from clearer operational definition. It is currently unclear whether this outcome is based on predefined clinical criteria, clinician judgement, or service-level decisions. Explicit criteria for success and re-presentation would improve objectivity and reproducibility.

In addition, while the planned quantitative analyses are comprehensive, further detail regarding handling of missing data, differential follow-up between pathways, and potential clustering effects across clinic sites would enhance the robustness of the analytical approach.

VERSION 1 - AUTHOR RESPONSE

Reviewer: 1

Comment 1

First, the protocol refers to the use of “boothless hearing tests” as part of the remote diagnostic pathway, yet no information is provided regarding the specific technology used, its validation status, or its agreement with standard audiometric testing performed in sound-treated environments. Given the central role of hearing assessment in ENT care, clearer description of the measurement methods and supporting evidence for their clinical reliability is essential.

Reply 1

Thank you for this comment. We have now clarified the audiometric equipment used in the remote pathway. Audiometric testing will be performed using calibrated boothless audiometers developed by hearX Group. These devices are commercially available systems that meet the required regulatory and calibration standards for clinical use. We have updated the manuscript to include their specifications/regulatory standards: “Audiometric testing will be performed using calibrated boothless audiometers developed by hearX Group (Pretoria, Gauteng, South Africa). These devices are commercially available systems that meet the required regulatory and calibration international standards for clinical use (ISO 8253-1/ ISO 389 Series/ IEC 60645-1/ ANSI S3.6) and are CE-marked registered and certified in Europe. The company’s regulatory standards are available in detail in their webpage: https://www.hearxgroup.com/products/heartest.”

This study is not designed to evaluate the diagnostic accuracy or non-inferiority of boothless audiometry compared with conventional sound-treated booth testing. Rather, the audiometric

equipment forms part of an established clinical pathway that is already in routine use within the Trust. Device selection and procurement are determined through standard organisational processes and are independent of the research team.

Comment 2

Second, although patients are not randomised to the remote or traditional pathways, the manuscript states that allocation based on clinical priority and waiting time will not introduce selection bias. In practice, these factors are likely to be associated with disease severity, comorbidity, and sociodemographic characteristics, which may influence both outcomes and service utilisation. Further explanation of how pathway allocation decisions are made, and how potential confounding will be addressed beyond propensity score weighting, would strengthen the methodological rigour.

Reply 2

Thank you for this important comment. Allocation to remote or face-to-face clinics is determined operationally by the Trust and is not based on patient demographics, disease severity or clinical characteristics. All referrals are clinically triaged for urgency only, and patients are then allocated sequentially to available clinic slots (remote or face-to-face) by administrative staff until each clinic is filled.

The clinics included in this study manage non-cancer ENT referrals; cancer referrals follow a separate two-week-wait pathway and are therefore not eligible for inclusion. Clinicians delivering care are not involved in the allocation of patients to clinic type.

While we acknowledge that unmeasured confounding may still exist in any observational study, the allocation process itself is not designed to preferentially direct particular patient groups to either pathway. To further address potential confounding, we will estimate propensity scores and apply propensity score weighting using logistic regression, as now described in the revised manuscript.

Comment 3

Third, the primary outcome of "successful diagnosis and management remotely" would benefit from clearer operational definition. It is currently unclear whether this outcome is based on predefined clinical criteria, clinician judgement, or service-level decisions. Explicit criteria for success and re-presentation would improve objectivity and reproducibility.

Reply 3

We have revised the manuscript to provide an explicit operational definition of the primary outcome. "Successful remote management" is now defined as a patient for whom a diagnosis and initial management plan are reached following a single remote consultation, without the need for additional appointments to establish the diagnosis.

Follow-up appointments undertaken solely to review investigation results or to support treatment planning do not constitute failure of remote management. Patients requiring further appointments in order to establish a diagnosis are classified as not successfully managed remotely.

Comment 4

In addition, while the planned quantitative analyses are comprehensive, further detail regarding handling of missing data, differential follow-up between pathways, and potential clustering effects across clinic sites would enhance the robustness of the analytical approach.

Reply 4

Missing data will be described in terms of proportion, patterns and likely mechanisms. Where appropriate, missing data will then be handled using multiple imputation, and this approach is now described in the revised manuscript.

Differences in follow-up duration between pathways will be addressed using time-to-event methods, including Cox proportional hazards models, to account for variation in time to definitive treatment or decision.

This study is currently being conducted within a single NHS Trust. As such, there is limited scope for analysing clustering effects across multiple sites; this has been clarified in the manuscript.

VERSION 2 - REVIEW

Reviewer 1

Name Fu, Xinxing

Affiliation Beijing Tongren Hospital, Beijing Institute of Otolaryngology

Date 01-Mar-2026

COI

Thanks for addressing all my concerns from last review. Now I have no further revision requests.
